# Halve the dose while maintaining image quality in paediatric Cone Beam CT

**DOI:** 10.1038/s41598-019-41949-w

**Published:** 2019-04-02

**Authors:** Anne Caroline Oenning, Ruben Pauwels, Andreas Stratis, Karla De Faria Vasconcelos, Elisabeth Tijskens, Annelore De Grauwe, Dimitra research group, Catherine Chaussain, Hilde Bosmans, Ria Bogaerts, Constantinus Politis, Laura Nicolielo, Guozhi Zhang, Myrthel Vranckx, Anna Ockerman, Sarah Baatout, Niels Belmans, Marjan Moreels, Mihaela Hedesiu, Pirsoka Virag, Mihaela Baciut, Maria Marcu, Oana Almasan, Raluca Roman, Ioan Barbur, Cristian Dinu, Horatiu Rotaru, Lucia Hurubeanu, Vlad Istouan, Ondine Lucaciu, Daniel Leucuta, Bogdan Crisan, Loredana Bogdan, Ciprian Candea, Simion Bran, Grigore Baciut, Reinhilde Jacobs, Benjamin Salmon

**Affiliations:** 1 Paris Descartes University - Sorbonne Paris Cité, EA 2496 - Orofacial Pathologies, Imaging and Biotherapies Lab, Montrouge, France and Dental Medicine Department - Bretonneau Hospital, HUPNVS, AP-HP, Paris, France; 2 Division of Oral Radiology, Faculdade São Leopoldo Mandic, Instituto de Pesquisas São Leopoldo Mandic, Campinas, Sao Paulo Brazil; 3 Department of Mechanical Engineering, Catholic University of Leuven, Leuven, Belgium; 4 Department of Radiology, Faculty of Dentistry, Chulalongkorn University, Bangkok, Thailand; 5 OMFS IMPATH research group, Department of Imaging and Pathology, Faculty of Medicine, University of Leuven and Oral & Maxillofacial Surgery, University Hospitals Leuven, Leuven, Belgium; 6 Department of Dental Medicine, Karolinska Institutet, Stockholm, Sweden; 7 Radiobiology Unit, Laboratory of Molecular and Cellular Biology, Institute for Environment, Health and Safety, Belgian Nuclear Research Centre, SCK•CEN, Boeretang200, 2400 Mol Belgium; 8 Department of Oral Radiology, Faculty of Dentistry, ‘Iuliu Hatieganu’ University of Medicine and Pharmacy, Cluj-Napoca, Romania

**Keywords:** Paediatric research, Risk factors

## Abstract

Cone beam CT (CBCT) for dentomaxillofacial paediatric assessment has been widely used despite the uncertainties of the risks of the low-dose radiation exposures. The aim of this work was to investigate the clinical performance of different CBCT acquisition protocols towards the optimization of paediatric exposures. Custom-made anthropomorphic phantoms were scanned using a CBCT unit in six protocols. CT slices were blinded, randomized and presented to three observers, who scored the image quality using a 4-point scale along with their level of confidence. Sharpness level was also measured using a test object containing an air/PMMA e,dge. The effective dose was calculated by means of a customized Monte Carlo (MC) framework using previously validated paediatric voxels models. The results have shown that the protocols set with smaller voxel size (180 µm), even when decreasing exposure parameters (kVp and mAs), showed high image quality scores and increased sharpness. The MC analysis showed a gradual decrease in effective dose when exposures parameters were reduced, with an emphasis on an average reduction of 45% for the protocol that combined 70 kVp, 16 mAs and 180 µm voxel size. In contrast, both “ultra-low dose” protocols that combined a larger voxel size (400 µm) with lower mAs (7.4 mAs) demonstrated the lowest scores with high levels of confidence unsuitable for an anatomical approach. In conclusion, a significant decrease in the effective dose can be achieved while maintaining the image quality required for paediatric CBCT.

## Introduction

The use of cone beam CT (CBCT) for dentomaxillofacial diagnosis has been growing substantially in conjunction with the concern regarding undetermined risks of the low-dose radiation exposures, especially for children and adolescents^1^. Despite the well-known higher radiosensitivity of paediatric patients, several indications have been described on how CBCT can positively impact the diagnosis and treatment outcomes^2^. In this way, the DIMITRA project (Dentomaxillofacial paediatric IMaging: an Investigation Towards low dose RAdiation induced risks - www.dimitra.be) aims to define the appropriate balance between dose and image quality in an age- and indication-oriented way, according to the recently introduced ALADAIP principle (As Low as Diagnostically Acceptable being Indication-oriented and Patient-specific)^2^.

The concept of image quality involves a number of variables, especially for three-dimensional modalities such as CBCT. In general, a better quality is achieved when the technical parameters of the unit are adjusted towards a high-resolution mode, which is often correlated with higher dose values. However, there is a noticeable difference between a high-quality or high-definition image and a diagnostically acceptable one for a given indication. On the other hand, if an exam is rejected for being of insufficient image quality, the principle of optimization is disregarded, since the patient will be re-exposed without any additional benefits^3^. To approach the optimized protocols, first, multiple CBCT scans may need to be repeated on *ex vivo* phantoms simulating as closely as possible the clinical conditions; then, the image quality may be evaluated depending on the setting applied and from a clinical point of view. The subjective image quality assessment is a method that seeks this balance (dose *vs* image quality) while it allows, to some extent, a clinical performance evaluation of a diagnostic imaging method. It is linked to the ability to evaluate imaging features without knowing the technical parameters applied on their acquisition and/or reconstruction. Therefore, the aim of this study was to provide data regarding subjective image quality of some pre-selected CBCT scan protocols obtained from recently reported age-specific anthropomorphic DIMITRA phantoms^4^. These data were also matched with objective measurements and accurate dose quantifications based on Monte Carlo (MC) simulation^5^ in order to provide optimization strategies in the paediatric dentomaxillofacial radiology field.

## Material and Methods

### Ethical implications

All the methods were carried out in accordance with relevant guidelines and regulations. The experimental protocols of the DIMITRA project were approved by local institutional board and have received the ethical Agreement Numbers 16-021 (Paris Descartes University) and B322201525196 (KU Leuven).

### DIMITRA Phantoms

The previously described DIMITRA phantoms were selected for this study^4^. These phantoms were obtained by means of covering six paediatric skulls (age range of 4 to 10 years-old) coming from the anatomical collection of the University of Hasselt (Hasselt, Belgium) with a soft tissue substitute (Mix-D)^6^ in order to simulate the human soft tissues and their effects on x-ray attenuation, scattering and the resulting images.

### Pilot study: CBCT and Monte Carlo simulation

A pilot study took place in order to pre-investigate which protocols would be more suitable for subjective image quality assessment in paediatric patients. Whereas some CBCT machines provide some non-changeable default protocols, the unit used in the present study (CS9300, Carestream, Rochester, NY, USA) allows several combinations of technical parameters. For this reason, one DIMITRA phantom was scanned with 24 different protocols, varying kVp, mAs, and voxel size. A Monte Carlo (MC) framework was used to calculate the effective dose (ED) for all those protocols (further information regarding MC simulation are presented in the following sections). From those data, 6 protocols applicable for the study itself were defined including the utmost protocols (higher and lower dose) as well as four intermediates protocols, at a lower level of mAs (Table 1). This selection was based on image quality overview, ED provided by MC simulation and in accordance with previous studies showing optimal results - dose reduction and acceptable image quality - at lower mAs^7,8^.

**Table 1:** Technical parameters of the acquisition protocols.

Protocol	Tube Voltage (kVp)	Tube Current (mA)	Exposure Time (s)	Scanning Time (s)	Number of Projections	Voxel size (µm)
P1	90	5	8	23	320	180
P2	90	2	8	23	320	180
P3	80	2	8	23	320	180
P4	70	2	8	23	320	180
P5	80	2	3.7	16	220	400
P6	70	2	3.7	16	220	400

CS9300 specifications: tube pre-filtration: 2.5 mm Al equivalent; gantry rotation angle: 220°; detector pixel size: 127 µm; pre-processing/reconstruction: filtered back projection/FDK.

### CBCT Scanning and images selection

According to the pilot data, the 6 DIMITRA phantoms were scanned with the CS9300 device under the 6 protocols described in Table 1, with a field of view of 8 × 8 cm.

All data were imported into MeVisLab image processing and visualization platform (MeVis Research, Bremen, Germany). For the evaluation of anatomical features linked to bone assessment and tooth assessment (Table 2), 48 representative regions were selected on the CBCT scans. Slice selection aimed to represent similarly the six phantoms, upper and lower jaws as well as anterior and posterior regions. A region of interest (ROI) was selected for each region/protocol and saved as new images in DICOM format (Digital Imaging and Communications in Medicine) to reduce computation time in the following step. To obtain exactly the same slice for all protocols in a given phantom and anatomical region, the volume stacks were registered by means of the spatial alignment of the correspondent ROIs, using protocol 1 (P1: higher spatial resolution and exposure factors) as the reference image.

**Table 2:** Approach for the subjective image quality assessment.

General observation task	Specific observation task	Question
Bone Assessment	Trabecular bone pattern	*Can you evaluate the trabecular bone pattern?*
	Cortical bone	Can you delineate the cortical outline of the anatomical landmark*?
Tooth Assessment	Enamel and Dentin	*Can you delineate the enamel and dentin?*
	Lamina dura and periodontal ligament space	*Can you distinguish the lamina dura and periodontal ligament space?*

*Anatomical landmarks included in the observations: maxillary sinus, nasal cavity, nasopalatine canal, mandibular canal, mental foramen and lingual foramen.

### Subjective image quality assessment

**Figure 1:**
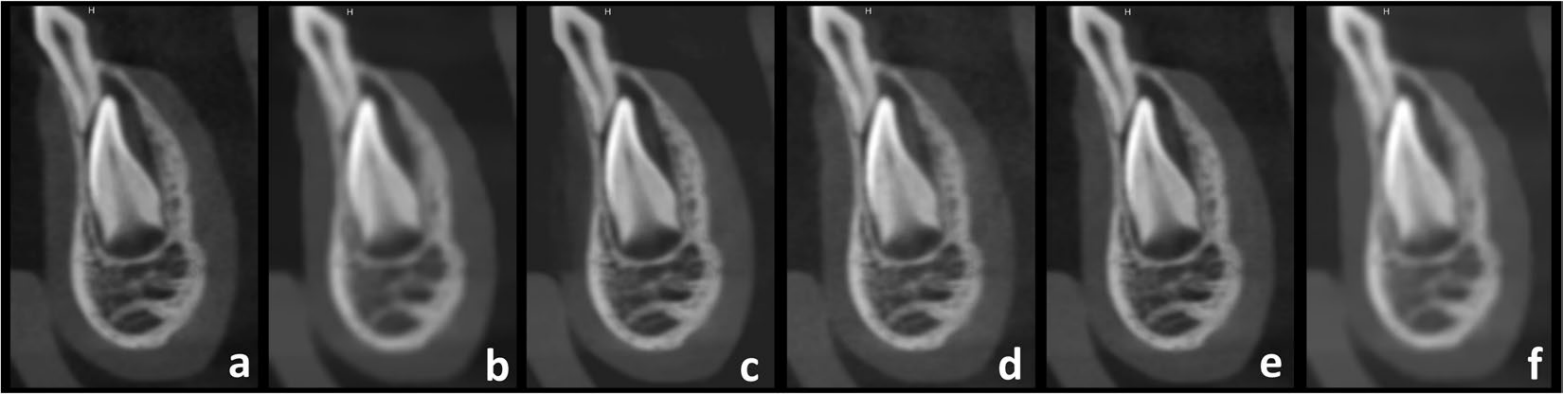
Representative set of slices randomly disposed for the observers. (**a**) Protocol 3, (**b**) Protocol 5, (**c**) Protocol 1, (**d**) Protocol 4, (**e**) Protocol 2, (**f**) Protocol 6.

**Figure 2:**
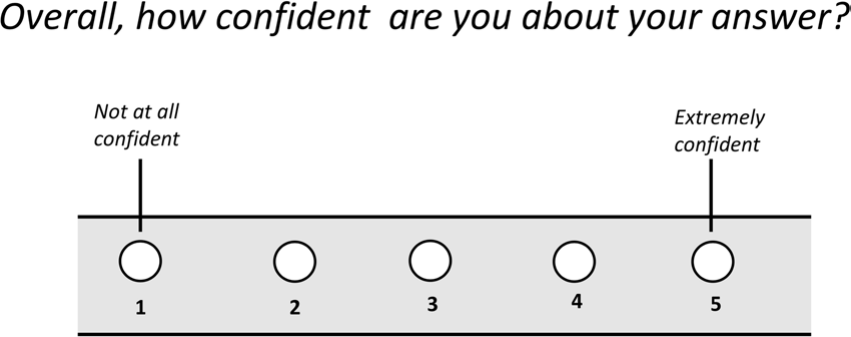
Visual analogue scale (VAS) adapted to indicate the observers’ level of confidence.

Three observers with expertise in dentomaxillofacial paediatric radiology were previously trained and calibrated regarding the observation method. Training, calibration and observation sessions were performed with the same medical monitor (Barco, Kortrijk, Belgium) and under standard conditions. The slices selected in a given region and phantom, but from the different acquisition protocols, were blinded and randomly displayed in the same screen (Fig. 1) to be evaluated at the same time by means of the specific questions presented in the Table 2. For each question, the observers examined 12 screens in successive with six slices each time, resulting in 288 answers that were given on a four-point scale: (1) I definitely cannot evaluate, (2) I cannot evaluate, (3) I can evaluate, (4) I definitely can evaluate. In addition, they pointed out in a visual analogue scale (VAS) how confident they were concerning the answers (Fig. 2). After 30 days, 25% of the images were re-assessed to obtain the intra- and inter-observer reproducibility data.

### Objective image quality assessment

The SEDENTEXCT IQ phantom was scanned under the same protocols performed for subjective assessment (Table 1). A single insert was used in this study, *i.e*. the “CT number – air” insert. It is a small polymethyl-methacrylate (PMMA) cylinder, approximately 3.45 cm in diameter and 2.0 cm in height, and contains a central air-filled cylinder of 1.0 cm diameter. It was placed centrally in the SEDENTEXCT IQ phantom; the rest of the phantom was half filled up with PMMA inserts in order to represent the attenuation of a child, according to a previous study^8^.

To determine the sharpness of the images, the edge between the air and PMMA was used to derive the edge spread function, from which the full width at half maximum (FWHM) was calculated through a Gaussian curve fitting^9^. Furthermore, the mean grey value (MGV) and standard deviation (SD) was measured for air and PMMA, and the contrast-to-noise ratio (CNR) was calculated as the difference in MGV between the two materials divided by the root sum of squares of the SDs.

### Dose calculations - Monte Carlo simulation

A fully validated Monte Carlo (MC) framework developed for the DIMITRA project was used for dosimetric calculations^10^. Scanner-specific input files were used to customize the framework according the CS9300 CBCT unit features (scanner-specific technical, geometric, and acquisition details). Firstly, absorbed organ doses (µGy) were calculated using the MC framework and 3 head and neck paediatric voxels models representing boys of 5, 8 and 10 years-old^5,11^. These ages were selected to be consistent with the age range of the DIMITRA anthropomorphic phantoms. Then, the radiation-induced risk was determined by estimating the ED, taking into account the organ-specific radiosensitivity weighting factors (applied over absorbed doses)^12^ and the fraction of any radiosensitive organ present in each phantom.

### Statistical analysis

Data were analyzed by means of descriptive statistics and Friedman ANOVA test. In addition, Dunn’s test was used to perform pairwise comparison of the assigned scores between protocols. Intra- and inter-observer reproducibility was assessed by means of Kappa statistic, taking Landis & Kock (1977) values as reference (0–0.19, poor agreement; 0.20–0.39, fair agreement; 0.40–0.59, moderate agreement; 0.60–0.79, substantial agreement; 0.80–1.00, almost perfect agreement)^13^. A commercially available software (Prism 5, GraphPad, San Diego, CA, USA) was used for data evaluation. The level of statistical significance was set at 0.05.

## Results

### 90 kVp to 70 kVp and 40 mAs to 16 mAs reductions do not significantly impair the subjective image quality

**Figure 3:**
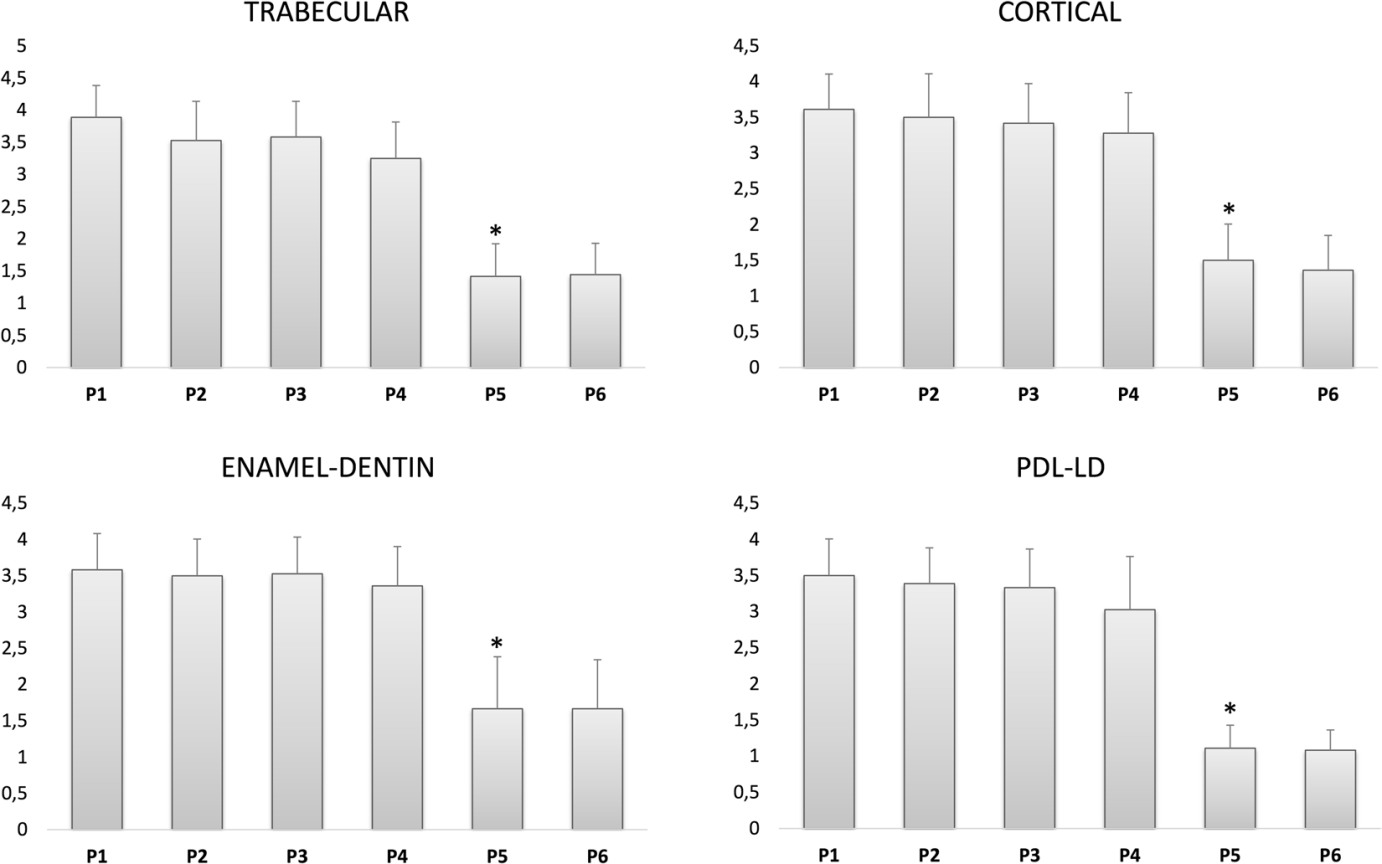
Mean values and standard deviations merged for all the observers for the protocols 1 to 6 related to the four anatomical parameters (trabecular bone, cortical bone, enamel and dentin, PDL-LD: periodontal ligament and lamina dura). *Significant inter-comparisons between protocols P5 and P1–P4, p < 0.05.

The descriptive analysis of the assigned scores, merged for all the observers inside a given protocol are presented in Fig. 3. The highest mean score values were concentrated in protocols P1 to P4, whereas the protocols P5 and P6 have shown mean score values mostly below 2.

A statistically significant difference among protocols was revealed by the Friedman ANOVA test for all the anatomical parameters (p < 0.0001). However, Dunn’s multiple comparison test demonstrated no statistically significant differences among protocols P1 to P4 (180 µm voxel size). On the other hand, these protocols (P1 to P4) were significantly different from P5 and P6 (400 µm voxel size; p < 0.05) (Table 3).

**Table 3:** Pairwise comparison between the protocols (Dunn’s test).

Dunn’s Multiple Comparison Test	Difference in rank sum			
	Trabecular bone pattern	Cortical Bone	Enamel Dentin	LD_PDL
P1 vs P2	25	6.5	6	6
P1 vs P3	21	12.5	4	10
P1 vs P4	44	23.5	16.5	28.5
P1 vs P5	131*	115.5*	114*	118*
P1 vs P6	130*	121*	114.5*	119.5*
P2 vs P3	−4	6	−2	4
P2 vs P4	19	17	10.5	22.5
P2 vs P5	106*	109*	108*	112*
P2 vs P6	105*	114.5*	108.5*	113.5*
P3 vs P4	23	11	12.5	18.5
P3 vs P5	110*	103*	110*	108*
P3 vs P6	109*	108.5*	110.5*	109.5*
P4 vs P5	87*	92*	97.5*	89.5*
P4 vs P6	86*	97.5*	98*	91*
P5 vs P6	−1	5.5	0.5	1.5

*Significant inter-comparisons, p < 0.05; LD_PDL: lamina dura-periodontal ligament space.

For intra-observer assessment, all scores were grouped; the agreement ranged from substantial to excellent (Observer 1 = 0.818, Observer 2 = 0.707, Observer 3 = 0.746). Table 4 shows the pairwise inter-observer agreement for all anatomical parameters grouped and separated. On average, we can observe that the enamel-dentin parameter showed the lowest agreement and the trabecular bone pattern the highest agreement. Generally, the agreement ranged from 0.5 to 0.7 (moderate to substantial), except for Observers 1 and 2 in enamel-dentin assessment (0.332 – fair agreement). Observers’ confidence scores were high (4 or more) for all protocols and anatomical parameters; a slight drop could be detected for the protocol P4, both in observer scores (Fig. 3) and confidence (Table 5).

**Table 4:** Inter-observer agreement (Kappa statistic).

	ALL		TRABECULAR		CORTICAL		E_D		LD_PDL	
	2	3	2	3	2	3	2	3	2	3
1	0.542	0.666	0.687	0.759	0.525	0.608	0.332	0.503	0.636	0.788
2	—	0.683	—	0.751	—	0.736	—	0.534	-	0.677

1, 2, 3: Observers; ALL: all anatomical parameters grouped (trabecular + cortical + E_D + LD_PDL); E_D: enamel, dentin; LD_PDL: lamina dura and periodontal ligament.

**Table 5:** Level of confidence indicated by the observers to attribute the image quality scores.

Protocol	VAS average data			
	Trabecular bone pattern	Cortical Bone	Enamel Dentin	LD_PDL
P1	4.86	4.56	4.47	4.47
P2	4.72	4.50	4.44	4.19
P3	4.67	4.44	4.33	4.17
P4	4.39	4.19	4.17	4.00
P5	4.72	4.64	4.33	4.94
P6	4.72	4.72	4.36	4.92

VAS: visual analogue scale.

The objective assessment obtained from FWHM values showed a decrease in sharpness (*i.e*. increasing FWHM values) from the protocols 1 to 6. The highest sharpness was found for those protocols obtained with smaller voxel size (P1 – 0.668; P2 – 0.702; P3 – 0.775; P4 – 0.798; P5 – 0.863; P6 – 0.826). By comparing within the same voxel size, CNR decreased from P1 to P4 (180 µm) and between P5 and P6 (400 µm) (P1 – 14.95; P2 – 11.07; P3 – 10.85; P4 – 9.25; P5 – 26.80; P6 – 21.33).

### Effective dose substantially decreases with kVp reduction, especially in young children

Table 6 presents the results for the effective doses (EDs) for the 3 paediatric models and for all performed scan protocols. The highest measured EDs were calculated for protocol P1, for the youngest model (5 years-old, 98 µSv). As the difference between P1 and P2 was a mAs decrease of 60% in P2, the same reduction (60%) could be linearly extrapolated for the effective dose, since the relation between mAs and ED follows a linear pattern. However, non-linear dose decreases were observed for P3 and P4, on which gradual reductions on beam energy were made (10 kVp), keeping the same level of 16 mAs. The ED ratio decrease is furthermore significant on those models simulating young children. For instance, P4 generates 63% less ED in 5 years-old and 58% in 8 years-old compared to P2.

**Table 6:** Effective doses (EDs) for the 3 paediatric models and for all performed scan protocols.

Protocol	Effective dose - ED (µSv)			% Reduction on ED		
	5 y-o	8 y-o	10 y-o	5 y-o	8 y-o	10 y-o
P1	98	70	58.7	−	−	−
P2	39.2	28	23.5	60	60	60
P3	27.8	20.5	17.5	29.08	26.78	25.53
P4	14.6	11.7	9.8	47.48	42.92	44
P5	12.8	9.5	8.1	12.32	18.8	17.34
P6	6.7	5.4	4.5	47.65	43.15	44.44

### Voxel size is a key point in image quality vs dose balance

The most prominent data refers to the ED average reduction of 45% for P4 in comparison to P3 (Table 6), considering the absence of statistical differences on the observations scores (Fig. 3). A slight reduction on the ED could be detected between the protocols P4 (180 µm voxel size) and P5 (400 µm) (16% on average), against the remarkable decrease on image quality scores (averages are mostly under 1.6 – Fig. 3). Despite the noticeable difference on ED between P5 and P6 (45%; the same as P3 vs. P4), the clinical implication is questionable considering the low performance of these protocols to adequately show the anatomical features (scores are mostly under 1.6).

## Discussion

Results of this research indicate that it is possible to significantly decrease radiation dose by means of technical parameter reduction, while keeping the clinical performance for paediatric diagnostic tasks, at a given level and for the CBCT model used in this study. However, “ultra low-dose” protocols, combining a low mAs and an increased voxel size, are not acceptable in relation to image quality and further diagnosis. Indeed, such protocols have shown much lower scores for all parameters, associated with a high level of confidence indicated by the observers regarding their rejection decision. This poor performance is probably related to the decrease in sharpness in the larger voxel size option demonstrated by the higher values for FWHM. We believe this objective parameter (FWHM–sharpness) the most suitable to link with subjective assessment data rather than contrast-to-noise ratio (CNR) taking into account that the last is more relevant when the voxel size is fixed. Smaller voxel size usually provides higher sharpness and better clinical performance but lower CNR due to the higher noise^14^. In that sense, CNR was not a good predictor for a clinically acceptable/unacceptable image. Therefore, it is difficult to directly connect research tools (*i.e*. objective image quality measurements) to clinical image quality, implying that optimization is a wider concept and a challenging task. Several key-points should be taken into account before the choice of a CBCT protocol; patient features like age, size and gender, as well as the specific exam indication must be always considered and weighted towards a good balance between benefit and radiation risk^2,15^.

Studies involving clinical imaging analysis are difficult to delineate and manage. For obvious ethical reasons, *in vivo* studies conducted with variations of protocols and exposure factors are not acceptable, especially for paediatric patients. However, the commercially available experimental phantoms are mostly made with adult skulls and developed for students training or for dosimetric proposal. These phantoms are covered by a soft tissue simulation material capable to simulate x-ray attenuation but with resulting images often presenting an increasing noise in comparison with *in vivo* CBCT exams. For this reason, in the present research, phantoms were custom made using paediatric natural skulls covered by Mix-D, a material that simulates soft tissues and able to fit on the bone surfaces without gaps or excessive infiltration in the cancellous bone or cavities^2^. The resulting CBCT images have shown bone covering and tomographic density quite similar to the CBCT appearance of human soft tissues. In addition, six paediatric skulls with different age-ranges were used. This strategy allows the evaluation of non-dependent additional variables like head size, age and dental formation stage.

In the present study, we have opted for the subjective assessment of standardized and registered slices selected in pre-defined regions. Although this strategy does not fully reproduce the clinical practices of CBCT interpretation (*i.e*. dynamic scrolling assessment in a native or viewer software), it allows the evaluation in a more controlled and standardized manner, avoiding to some extent, the “observer approach variability”. Furthermore, it has been shown that both methods are useful tools for subjective image quality assessment^16,17^.

The restriction of the Field of view (FOV) to the region of interest seems to be the most efficient strategy for dose reduction, appearing as a relevant source of optimization^14,18^. For this DIMITRA task, the field of 8 × 8 cm was chosen, given that it is a relatively small FOV and is capable of including both jaws of a child in a single scan. Additionally, the present results have shown that it is possible to achieve a good balance between dose and image quality using this FOV, and even small details can be assessed in likely optimized protocols (e.g. evaluation of lamina dura and periodontal ligament space). However, it is worth mentioning that greater FOV restrictions (*e.g*. 5 × 5 cm) must be used for some specific indications favoring both dose reduction and imaging quality improvement^2^.

Whereas the protocol P4 (70 kVp, 16 mAs, 180 µm of voxel size) can be considered the optimal one (acceptable observers’ scores at lower exposure factors), we should not ignore the slight drop both in observer scores and confidence level (VAS), which was consistently seen for all anatomical parameters. However, ED calculations via MC simulations have shown a reduction by almost half in the P4 dose in comparison to P3, supporting the choice of P4 mainly for younger children.

An early age of the anthropomorphic phantoms (5 years-old), an intermediate age (8-years-old) and the older one (10 years-old) were chosen to calculate the doses by MC simulations, aiming a consistency between the image quality assessment and dosimetry. In general, an average increase of 36% (32–40%) in the effective doses was detected in the younger age (5 years-old) in comparison to the older (10 years-old), markedly for higher dose protocols (P1 and P2 – 40% of increase). In addition, voxels phantoms of male children were chosen considering that MC calculations demands plenty computational time and workflow. Male/female MC simulations were subject of previous studies of our group^5,18^ and it was already demonstrated that doses were slightly lower in males than females. Despite this slight disparity, difference in organ doses (+/−5%) could be considered within the statistical uncertainty of the MC dose calculation^5^. However, results of the present and previous studies^5^ emphasize the need of age-specific voxels models, as large dose differences were calculated among the simulations performed at the same exposure conditions using models of different ages.

It is important to stress that the optimization strategies suggested here can be applied for one specific CBCT unit (CS9300). For instance, as the mAs reduction is more dose efficient than a kVp reduction, we can try reducing mAs even more than the lower limit of CS9300, keeping or not, “diagnosticable” images. There are many CBCT devices currently in the market, allowing a number of variations in technical and exposure factors. The range of effective radiation dose delivered by those devices is wide (around 10 to 1200 µSv), while the image quality varies drastically within and between CBCT units^19–21^. For this reason, the protocols must be carefully evaluated and chosen according to the diagnostic needs, imaging requirements and patient features. If the CS9300 unit allows to widely adjust the tube voltage and current, the voxel size remains fixed by the reconstruction process. Namely, intermediate voxel sizes, between 180 µm (P4) and 400 µm (P5), cannot be selected, hence evaluated. Also, voxel size selection should be indication-specific. For instance, autotransplantation with replica printing requires a different parameter set-up than a diagnostic task for dental trauma^2,18^. Moreover, even though reconstruction is based on Feldkamp, David, Kress (FDK) algorithm in current commercially available CBCT units, iterative approaches may be promising methods to reduce the required projections, thus the dose, while maintaining the image quality^22–24^. Further studies are required to overcome these limitations and take into account the constant technical evolutions. In this context, the present results emphasize these needs and possibilities towards optimization according to ALADA and ALADAIP principles^2,3^.

Based on phantoms, the present study did not consider the negative effect of the potential patient motion, a daily issue especially with children, resulting in blurring that harms the images reading. To limit motion artefacts, selection of fast scan protocols, implying reduced time of exposure and/or number of projections, should be balanced with the required image quality according to the ALADAIP principle^2^. Recently, methods for detection and correction of motion artefacts have been proposed^25,26^; undoubtedly these are promising tools towards optimization strategies.

In conclusion, the results of this research task highlight the possibility to achieve a considerable decrease in the effective dose, while keeping the required image quality for paediatric CBCT diagnostics. The protocol P4, combining relatively low mAs (16) and kVp (70) with a small voxel size (180 µm) seems to be the optimal option under the tested conditions, due to the low effective dose associated to high image quality scores. Therefore, there is a pressing need for indication-oriented optimization in the paediatric diagnostic field, considering the diagnostic needs and specific image requirements together with age and gender data.
