# Publisher Correction: Halve the dose while maintaining image quality in paediatric Cone Beam CT

**DOI:** 10.1038/s41598-020-59352-1

**Published:** 2020-02-07

**Authors:** Anne Caroline Oenning, Ruben Pauwels, Andreas Stratis, Karla de Faria Vasconcelos, Elisabeth Tijskens, Annelore de Grauwe, Dimitra research group, Catherine Chaussain, Hilde Bosmans, Ria Bogaerts, Constantinus Politis, Laura Nicolielo, Guozhi Zhang, Myrthel Vranckx, Anna Ockerman, Sarah Baatout, Niels Belmans, Marjan Moreels, Mihaela Hedesiu, Pirsoka Virag, Mihaela Baciut, Maria Marcu, Oana Almasan, Raluca Roman, Ioan Barbur, Cristian Dinu, Horatiu Rotaru, Lucia Hurubeanu, Vlad Istouan, Ondine Lucaciu, Daniel Leucuta, Bogdan Crisan, Loredana Bogdan, Ciprian Candea, Simion Bran, Grigore Baciut, Reinhilde Jacobs, Benjamin Salmon

**Affiliations:** 1 Paris Descartes University - Sorbonne Paris Cité, EA 2496 - Orofacial Pathologies, Imaging and Biotherapies Lab, Montrouge, France and Dental Medicine Department - Bretonneau Hospital, HUPNVS, AP-HP, Paris, France; 2 Division of Oral Radiology, Faculdade São Leopoldo Mandic, Instituto de Pesquisas São Leopoldo Mandic, Campinas, Sao Paulo Brazil; 3 Department of Mechanical Engineering, Catholic University of Leuven, Leuven, Belgium; 4 Department of Radiology, Faculty of Dentistry, Chulalongkorn University, Bangkok, Thailand; 5 OMFS IMPATH research group, Department of Imaging and Pathology, Faculty of Medicine, University of Leuven and Oral & Maxillofacial Surgery, University Hospitals Leuven, Leuven, Belgium; 6 Department of Dental Medicine, Karolinska Institutet, Stockholm, Sweden; 7 Radiobiology Unit, Laboratory of Molecular and Cellular Biology, Institute for Environment, Health and Safety, Belgian Nuclear Research Centre, SCK•CEN, Boeretang200, 2400 Mol, Belgium; 8 Department of Oral Radiology, Faculty of Dentistry, ‘Iuliu Hatieganu’ University of Medicine and Pharmacy, Cluj-Napoca, Romania

Correction to: *Scientific Reports* 10.1038/s41598-019-41949-w, published online 02 April 2019

The original version of this Article contained an error in the order of author names, which were incorrectly listed as ‘Anne Caroline Oenning’, ‘Ruben Pauwels’, ‘Andreas Stratis’, ‘Karla De Faria Vasconcelos’, ‘Elisabeth Tijskens’, ‘Annelore De Grauwe’, ‘Reinhilde Jacobs’, ‘Benjamin Salmon’ & ‘Dimitra research group’.

This error has now been corrected in the HTML and PDF versions of this Article

